# CK7 and consensus molecular subtypes as major prognosticators in ^V600E^*BRAF* mutated metastatic colorectal cancer

**DOI:** 10.1038/s41416-019-0560-0

**Published:** 2019-09-02

**Authors:** Fotios Loupakis, Paola Biason, Alessandra Anna Prete, Chiara Cremolini, Filippo Pietrantonio, Nicoletta Pella, Emanuela Dell’Aquila, Elisa Sperti, Clizia Zichi, Rossana Intini, Vincenzo Dadduzio, Marta Schirripa, Francesca Bergamo, Carlotta Antoniotti, Federica Morano, Francesco Cortiula, Giovanna De Maglio, Lorenza Rimassa, Valeria Smiroldo, Lorenzo Calvetti, Giuseppe Aprile, Lisa Salvatore, Daniele Santini, Giada Munari, Roberta Salmaso, Vincenza Guzzardo, Claudia Mescoli, Sara Lonardi, Massimo Rugge, Vittorina Zagonel, Massimo Di Maio, Matteo Fassan

**Affiliations:** 1 Department of Oncology, Veneto Institute of Oncology IOV—IRCCS, Padua, Italy; 2 Department of Translational Research and New Technologies in Medicine and Surgery, University of Pisa, Pisa, Italy; 3 Department of Medical Oncology, Fondazione IRCCS Istituto Nazionale dei Tumori, Milan, Italy; 4 Department of Oncology and Hemato-oncology, University of Milan, Milan, Italy; 5 Department of Oncology, University and General Hospital, Udine, Italy; 6 Department of Medical Oncology, Campus Bio-Medico University of Rome, Rome, Italy; 7 Department of Oncology, University of Turin at Umberto I “Ordine Mauriziano” Hospital, Turin, Italy; 8 Department of Medicine (DAME), University of Udine, Udine, Italy; 9 Department of Pathology, University Hospital of Udine, Udine, Italy; 10 Medical Oncology and Hematology Unit, Humanitas Cancer Center, Humanitas Clinical and Research Center—IRCCS Rozzano, Milan, Italy; 11 Department of Oncology, General Hospital San Bortolo, Unità Locale Socio-Sanitaria 8 Berica, Vicenza, Italy; 12 Unit of Oncology, Polyclinic GB Rossi, AOUI, Verona, Italy; 13 Surgical Pathology & Cytopathology Unit, Department of Medicine (DIMED), University of Padua, Padua, Italy; 14 Present Address: U.O.C Oncologia, Fondazione Policlinico Universitario Agostino Gemelli IRCCS, Roma, Italy

**Keywords:** Colorectal cancer, Prognostic markers

## Abstract

**Background:**

^V600E^*BRAF* mutated metastatic colorectal cancer (mCRC) is a subtype (10%) with overall poor prognosis, but the clinical experience suggests a great heterogeneity in survival. It is still unexplored the real distribution of traditional and innovative biomarkers among ^V600E^*BRAF* mutated mCRC and which is their role in the improvement of clinical prediction of survival outcomes.

**Methods:**

Data and tissue specimens from 155 ^V600E^*BRAF* mutated mCRC patients treated at eight Italian Units of Oncology were collected. Specimens were analysed by means of immunohistochemistry profiling performed on tissue microarrays. Primary endpoint was overall survival (OS).

**Results:**

CDX2 loss conferred worse OS (HR = 1.72, 95%CI 1.03–2.86, *p* = 0.036), as well as high CK7 expression (HR = 2.17, 95%CI 1.10–4.29, *p* = 0.026). According to Consensus Molecular Subtypes (CMS), CMS1 patients had better OS compared to CMS2-3/CMS4 (HR = 0.37, 95%CI 0.19–0.71, *p* = 0.003). Samples showing less TILs had worse OS (HR = 1.72, 95%CI 1.16–2.56, *p* = 0.007). Progression-free survival analyses led to similar results. At multivariate analysis, CK7 and CMS subgrouping retained their significant correlation with OS.

**Conclusion:**

The present study provides new evidence on how several well-established biomarkers perform in a homogenous^V600E^*BRAF* mutated mCRC population, with important and independent information added to standard clinical prognosticators. These data could be useful to inform further translational research, for patients’ stratification in clinical trials and in routine clinical practice to better estimate patients’ prognosis.

## Background

^V600E^*BRAF* mutation is detected in 8–12% of colorectal cancer (CRC) patients, accounting for more than 90% of CRC *BRAF* mutations.^1^ It is an independent negative prognostic factor in CRC across all stages.^2,3^ Furthermore, a recent consensus work identified *BRAF* mutational status as one of the top five fundamental stratification characteristics in the initial evaluation of metastatic CRC (mCRC) patients, together with *RAS* mutations, patients’ performance status, primary tumour sidedness and presence of liver-limited disease.^4^

Despite the evidence of its prognostic significance, great heterogeneity in survival outcome is evident among ^V600E^*BRAF* mutated mCRC.^5^ Indeed, some patients with ^V600E^*BRAF* mutated mCRC may experience prolonged survival and durable response to therapies, while other patients develop rapid resistance.^6^ These observations led to the hypothesis that a better stratification based on clinical and molecular features should be explored when considering ^V600E^*BRAF* mCRC as a separate disease. To achieve this goal, correct methodology, homogeneous patients’ cohorts and adequate sample size are of crucial importance. We recently proposed a clinical risk score prognostic calculator based on ECOG PS, tumour grading, presence of liver metastases, presence of lung metastases and presence of nodal involvement, CA19.9, CEA, LDH levels and neutrophils/lymphocytes ratio.^7^ Moreover, a ‘simplified’ version based only on the first five covariates was subsequently developed as functional and reliable tool for multivariate modelling of translational analyses.

Caudal type homeobox 2 (*CDX2*) is a gene encoding a protein involved in cell differentiation, adhesion and polarity. It has been hypothesised that ^V600E^*BRAF* mutation and loss of CDX2, which are significantly associated, might cooperate in promoting CRC tumorigenesis.^8,9^ Dalerba et al. demonstrated that loss of CDX2 expression may confer poor prognosis to stage II-III CRC patients.^10^ Notwithstanding, no or limited information regarding the relative impact of *BRAF* mutations and other prognostic features were available.^11^

CRC has been classically associated to a CK20-positive and CK7-negative profile.^12,13^ Literature data suggest that among ^V600E^*BRAF* mutated CRC, a higher prevalence of CK20-negative tumours may be found.^14,15^

Most importantly, up to 30% of ^V600E^*BRAF* mutated cases show microsatellite instability (MSI). In early stages, ^V600E^*BRAF* mutated microsatellite stable (MSS) CRC have a poorer prognosis^16–18^;conversely, the prognostic impact of microsatellite instability-high (MSI-H) status in ^V600E^*BRAF* mutated patients is still debated. Venderbosch et al.^2^ retrospectively analysed a large cohort of 3063 patients from four different studies aiming to describe mutual influence on prognosis of microsatellite instability in *BRAF* mutated stage IV CRC and vice-versa. The prognostic influence of ^V600E^*BRAF* mutation in MSS was confirmed, but other definitive conclusions were limited by excessive subgrouping. Another retrospective study including only 14 ^V600E^*BRAF* mutated patients out of 55 MSI-H cases suggested a negative impact of ^V600E^*BRAF* mutation in MSI-H patients, but again small sample size limited any reliable consideration on the prognostic impact of microsatellite instability among ^V600E^*BRAF* mutated patients.^19^

A remarkable step forward in the description of CRC heterogeneity has been made by the Consensus Molecular Subtypes (CMS).^20^ The majority of ^V600E^*BRAF* (up to 70%) are classified into CMS1 subgroup, while 7 and 17% are grouped in CMS2-3 and CMS4, respectively. This heterogeneous distribution supports the rationale for exploring the prognostic relevance of CMS subgrouping among ^V600E^*BRAF* mCRC.

Another active field of interest in the definition of mCRC prognosis is the presence of tumour infiltrating lymphocytes (TILs). So far, no specific studies are available in literature concerning their role in ^V600E^*BRAF* mutated tumours.

Finally, Barras et al. categorised ^V600E^*BRAF* mutated CRC into two groups based on gene expression signatures: BM1 patients, accounting for approximately one third of cases, show activation of KRAS/mTOR/AKT/4EBP1 pathway, while BM2 group is characterised by dysregulation in the cell-cycle.^5^

Given the above reported assumptions, the aim of our work is to investigate the prognostic role of the most important and biologically sound prognostic markers in a large set of ^V600E^*BRAF* mutated mCRC patients, in order to better explain the wide inter-patient heterogeneity observed in routine clinical practice.

## Methods

Clinical and molecular data of ^V600E^*BRAF* mutated mCRC patients referred to eight Italian Oncology Units between January 2005 and December 2016 were collected. In particular, for each patient data on demographic, tumour characteristics, 1st line systemic treatment, locally assessed RECIST1.1 response and survival were retrieved. The study received Ethics approval from the Coordinating centre, i.e. Istituto Oncologico Veneto IOV, Principal Investigator Dr Fotios Loupakis and was subsequently approved by each centre according to Italian national regulations code 2017/34. Cases were deemed eligible if clinical data and archival tissue either of primary tumour and/or metastases were available.

Available primary and/or metastatic formalin-fixed paraffin-embedded (FFPE) surgical samples were processed using the Galileo CK3500 Arrayer (www.isenet.it), a semiautomatic and computer-assisted Tissue microarray (TMA) platform. Tissue cores (3 cores per sample; 1 mm in diameter) were obtained from each primary and metastatic lesion, respectively. Small biopsy samples were processed separately. Immunohistochemical stainings were automatically performed using the Bond Polymer Refine Detection kit (Leica Biosystems, Newcastle Upon Tyne, UK) in the BOND-MAX system (Leica Biosystems) on 4 μm-thick sections. Primary antibodies, dilutions and scoring evaluation are available upon request. Specific methods and scoring systems for each marker are reported below.

### CDX2

CDX2 expressions values were defined according to H-score, defined as the aggregate of total percentage of tumour cells expressing CDX2 at each particular intensity level from 0, +1 (weak intensity), +2 (moderate intensity) or +3 (strong intensity). In brief, the H-score was defined as: (Percent of CDX2 1 + tumour cells multiplied by intensity of 1) + (Percent of CDX2 2 + tumour cells multiplied by intensity of 2) + (Percent of CDX2 3 + tumour cells multiplied by intensity of 3). Thus, this composite score can range from 0 (a tumour which is completely negative) to a maximum of 300 (a tumour in which all the cells feature a 3+ staining). CDX2 results were split in tertiles as follows: 0–24 (low expression), 25–120 (intermediate expression), 121–300 (high expression).

### Cytokeratins

Cytokeratin expression pattern was evaluated by CK7 and CK20 expression. CK7 expression was categorised in low (values 0–1) and high (values 2–3), whereas CK20 expression was indicated as negative (no expression) or positive (values 1, 2 or 3), according to staining intensity in more than 10% of cancer cells.

MSI-H status was defined in the absence of nuclear immunostaining for one of the couples MLH1/PMS2 or MSH2/MSH6 in tumour cell. The diagnostic performance of immunohistochemistry in identifying MSI-H cases was tested by microsatellite analysis (Titano kit, Diatech Pharmacogenetics) in a series of 20 MMRd and 20 MMRp tumours.^21^

### Consensus molecular subtypes

CMS were assigned by assessing four IHC markers (FRMD6, ZEB1, HTR2B, CDX2) in combination with pan-cytokeratin (KER) to normalise results as reported in literature.^22^ Primary tumours and/or metastasis were then categorised into the 3 CMS classes (CMS1, CMS2/3 or CMS4) using the online classification tool (https://crcclassifier.shinyapps.io/appTesting). As previously described, MSI status was first used to define patients which belong to the CMS1 subtype.^22,23^

### Tumour infiltrating lymphocytes

Presence of TILs was evaluated on haematoxylin and eosin (H&E) stained slides and dichotomised by using a cut-off of 2.0: low number of TILs for tumours showing an average number of TILs <2.0, high number of TILs for tumours with ≥2.0 TILs.^24^

### BM1 and BM2 subgroups

To stratify tumours according to Barras et al.^5^ in BM1 and BM2 groups, we exploratively categorised each tumour based on the presence/absence of these five markers: CDK1, ATM, Phospho-Akt (Ser473), Cyclin D1 and Phospho-4E-BP1 (Thr70). Since BM1 is characterised by activation of PI3K/mTOR/AKT pathway, while BM2 of cell cycle pathway we assigned samples to BM1 or BM2 based on the coherence of the following parameters. Overexpression of Phospho-Akt, Phospho-4E-BP1, ATM and Cyclin D1 and downregulation of CDK1 were consistent with a BM1 profile. On the other hand, BM2 cases were characterised by overexpression of CDK1 and downregulation of the remaining markers. A tumour was considered positive in ATM if >10% of tumour cells were positive for nuclear ATM staining. The activation of the AKT/4E-BP1 cascade was defined in the presence of high expression levels of the phosphorylated forms of AKT and/or 4E-BP1. High levels of Cyclin D1 and CDK1 expression were defined in the presence of at least 50% of cancer cells positive (Cyclin D1 in the nucleus, CDK1 both in the nucleus and cytoplasm). Samples with 4 or 5 coherent parameters were defined as BM1 or BM2, whereas tumours in which 3 out of 5 parameters were coherent with the hypothesis were defined as borderline BM1 or BM2. Tumours with only 1 or 2 parameters coherent with the original classification were defined as not evaluable.

### Clinical score

Simplified score for estimating the prognostic impact of major clinical and pathological was calculated considering 5 parameters as previously described^7^: grading, ECOG PS at diagnosis of metastatic disease and sites of metastases at diagnosis (liver, lung, nodes). To calculate the score the following criteria were applied: ECOG PS 0 = 0 points; ECOG PS 1 = 2 points; ECOG PS 2–3 = 3 points. Tumour grading 1 or 2 = 0 points, tumour grading 3 or 4 = 1 point. Presence of liver metastases = 1 point; presence of lung metastases = 2 points, presence of distant nodes metastases = 2 points. The score is calculated as the total sum of points. Patients were classified as ‘low-risk’ if they had a score ranging from 0 to 2; they were classified as ‘intermediate-risk’ if the score was 3 or 4; they were classified as ‘high-risk’ if their score ranged from 5 to 9.

### Statistical analysis

The primary endpoint of the present analysis was Overall Survival (OS) for each variable analysed. OS was defined as time from metastatic disease diagnosis to death due to any cause. Secondary endpoints included: progression free survival (PFS) for each variable (PFS was defined as the time from 1st line treatment start date to 1st progression); the reproducibility of BM1/BM2 subgrouping and CMS distribution as assigned by means of IHC/TMA. For each determinant, comparison between samples from primary tumour and metastatic lesions was performed in order to explore their concordance.

Both OS and PFS and 95%CI were calculated using Kaplan–Meier method. Cox proportional Hazard model was adopted in the multivariate analysis including all covariates significantly correlated with survival in the univariate analysis.

PFS and OS were calculated in univariate analysis for the following molecular factors: CDX2, CK7, CK20 expression, CMS groups, BM1/BM2 groups and presence of TILs. Factors found significant at univariate analysis were included into the multivariate analysis for both OS and PFS including clinical score data as covariate.

## Results

A total of 155 patients were included. Males and females were equally represented (50.3%/49.7%, respectively). As expected, frequent features were: right-sidedness of primary tumour (74.2%), presence of synchronous metastases (65.8%), hepatic or nodal involvement (53 and 38% respectively), with 63% of patients having a single metastatic site at the time of stage IV disease diagnosis. A large proportion of patients had previous primary tumour resection (87.1%). Baseline characteristics and major clinical parameters are summarised in Table 1.

**Table 1:** Baseline^a^ characteristics and major clinical parameters

Characteristic	TOT = 155
	*N* (%)
Sex	
Female	77 (49.7%)
Male	78 (50.3%)
Age	
Median (range)	66 (28–85)
Age	
>70	55 (35.5%)
≤70	100 (64.5%)
Baseline ECOG PS	
0	112 (72.2%)
1	37 (23.9%)
≥2	6 (3.9%)
Primary tumour resected	
Yes	135 (87.1%)
No	20 (12.9%)
Primary tumour location	
Right	115 (74.2%)
Left	31 (20.0%)
Rectal	9 (6.8%)
Presentation of metastases	
Synchronous	102 (65.8%)
Metachronous	53 (34.2%)
Number of metastatic sites	
Single	97 (63%)
Multiple	57 (37%)
Missing	1
Sites of metastases at diagnosis	
Liver	82 (53%)
Lung	27 (17.4%)
Distant nodes	59 (38%)
Other	28 (18.1%)
Missing	1

^a^i.e. at the time of first-line treatment start or, for candidates to BSC only, at the first visit for metastatic disease

The vast majority of patients (89%) received at least one treatment for metastatic disease: first-line treatment was monochemotherapy ± a biologic agent (anti-EGFR monoclonal antibody or bevacizumab) in 9.4% of treated patients, doublet ± a biologic agent in 55.8%, triplet ± a biologic agent in 24.6%, immunotherapy in 7.3% and anti-BRAF treatment in 2.2%.

Distribution of molecular variables analysed in whole population is shown in Table 2, correlation data between paired single parameters are reported in Supplementary Table 1. For 46 patients, paired primary and metastasis samples were available: data obtained from IHC analyses were concordant in most cases, as shown in Supplementary Table 2.

**Table 2:** Distribution of molecular variables analysed in whole population

Characteristic	TOT = 155
	*N* (%)
CDX2	
Low	47 (32.6%)
Intermediate	50 (34.8%)
High	47 (32.6%)
*NE*^a^	*11*
CK7	
Low	81 (87.1%)
High	12 (12.9%)
*NE*	*9*
*Not tested*	*53*
CK20	
Low	11 (11.8%)
High	82 (88.2%)
*NE*^a^	*9*
*Not tested*	*53*
CMS	
1	44 (39.7%)
2–3	47 (42.3%)
4	20 (18.0%)
*NE*^a^	*44*
TILs	
Low	61 (39.6%)
High	93 (60.4%)
*NE*^a^	*1*
Barras et al. subtypes	
BM1	51 (49%)
BM2	53 (51%)
*NE*^a^	*51*
Clinical prognostic score^b^	
Low	69 (44.8%)
Intermediate	59 (38.3%)
High	26 (16.9%)
*NE*^a^	*1*

^a^Not evaluable

^b^Simplified version

After a median follow-up of 27.9 months (95%CI 20.3–35.5), 104 patients (67.1%) died. Median OS of the whole population was 18.5 months (95%CI 13.3–23.7), median PFS from the beginning of the first line treatment was 7.6 months (95%CI 5.2–10.0).

### Univariate analyses

**Fig. 1:**
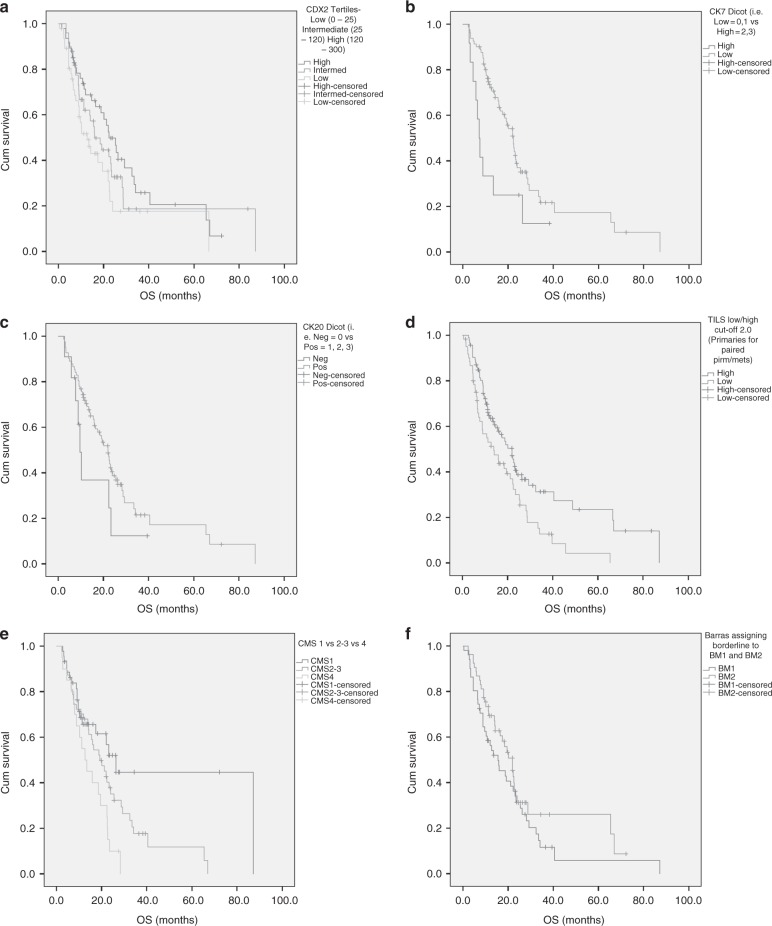
Kaplan–Meier curves for Overall survival (OS). **a** CDX2 tertiles expression (low vs intermediate and high). **b** CK7 expression (high vs low). **c** CK20 expression (negative vs positive). **d** TILs expression (low vs high). **e** CMS classification (CMS2/3 and CMS4 vs CMS1). **f** Barras classification (BM1 vs BM2)

Results on OS and PFS are reported in Table 3 and Supplementary Table 3 and graphically represented in Fig. 1 and Supplementary Fig. 1, respectively and described below for each single variable.

**Table 3:** Univariate analysis for overall survival

Characteristics	Median OS (months)	Overall survival		
		HR	95% CI	*p*
CDX2				
High	22.3	1	–	–
Intermediate	16	1.72	1.03–2.86	**0.036**
Low	12.7			
CK7				
Low	22.3	1	–	–
High	7.2	2.17	1.10–4.29	**0.026**
CK20				
Pos	22	1	–	
Neg	9.7	1.75	0.83–3.69	0.14
CMS				
1	26.3	1	–	–
2–3	19.2	2.7	1.41–5.26	**0.003**
4	12.7			
TILs				
High	22	1	–	–
Low	13.9	1.72	1.16– 2.56	**0.007**
BM				
2	22	1	–	–
1	15.6	1.37	0.87–2.17	0.177
Simplified score				
Low	23.3	1	–	–
Intermediate	19.5			
High	6.6	2.61	1.53–4.48	**<0.001**

Bold values indicate statistical significance *p* < 0.05

#### CDX2

Patients with low or intermediate expression had a shorter OS compared to patients with high expression (HR = 1.72, 95%CI 1.03–2.86, *p* = 0.036). Similar trend, but no significant differences were detected in terms of PFS (HR = 1.41, 95%CI 0.86–2.30, *p* = 0.169).

#### CK7–CK20

Patients with higher CK7 expression had a shorter OS compared to patients with lower CK7 expression (HR = 2.17, 95%CI 1.10–4.29, *p* = 0.026). No significant differences were detected in terms of PFS (HR = 1.13, 95%CI 0.56–2.29, *p* = 0.74). Patients with negative CK20 had a shorter OS compared to patients with positive CK20 (HR = 1.75, 95%CI 0.83–3.69, *p* = 0.14). Similar trend, but no significant differences were detected in terms of PFS (HR = 1.72, 95%CI 0.73–4.05, *p* = 0.21).

#### CMS

CMS2-3 or CMS4 patients had a shorter OS compared to CMS1 (HR = 2.70, 95%CI 1.41–5.26, *p* = 0.003), similar results were reported for PFS (HR = 2.22, 95%CI 1.14–4.35, *p* = 0.02).

#### TILs

Patients with low TILs levels had a shorter OS compared to patients with high levels (HR = 1.72, 95%CI 1.16–2.56, *p* = 0.007), results confirmed also in PFS (HR = 1.72, 95%CI 1.18–2.56, *p* = 0.005).

#### BM1/BM2

No significant differences between BM1 and BM2 patients were detected in terms of OS (HR = 1.37, 95%CI 0.87–2.17, *p* = 0.18), or PFS (HR = 1.27, 95%CI 0.80–2.03, *p* = 0.31).

#### Clinical score

As expected, patients with high score had a shorter OS compared to patients with intermediate or low score (HR = 2.61, 95%CI 1.53–4.48, *p* < 0.001), similar results were obtained in terms of PFS (HR = 2.13, 95%CI 1.21–3.75, *p* = 0.009).

### Multivariate analysis

Since the clinical score was built on OS data, it was included to adjust for integrating clinical and molecular prognostication. At multivariable analysis, CK7 overexpression was independently associated with worse OS with a HR of 2.11 (95%CI 1.03–4.34, *p* = 0.041), as was CMS2-3 and 4 over CMS1, with a HR of 2.22 (95%CI 1.03–5.02, *p* = 0.049). Complete data are reported in Table 4. The poor prognostic score that was determined clinically retained an independent prognostic impact (HR = 2.42, 95%CI 1.16–5.05, *p* = 0.019).

**Table 4:** Multivariate analysis for overall survival

Characteristics	Overall survival		
	HR	95%CI	*p*
CDX2			
High	1	–	–
Low + Intermediate	1.92	0.94–4.00	0.07
CK7			
Low	1	–	–
High	2.11	1.03–4–34	**0.041**
CMS			
1	1	–	–
2–3 + 4	2.22	1.03–5.02	**0.049**
TILs			
High	1	–	–
Low	1.19	0.66–2.17	0.55
Simplified score			
Intermediate + Low	1	–	–
High	2.42	1.16–5.05	**0.019**

Bold values indicate statistical significance *p* < .05

Similarly, in multivariate analysis for PFS, CMS2-3 and 4 were significant determinants of worse outcome over CMS1 (HR 2.17, 95%CI 1.01–4.76, *p* = 0.049). Complete data are reported in Supplementary Table 4

## Discussion

In the present study, we explored and clarified the prognostic role of CDX2, CK7 and 20, TILs, CMS and BM1/BM2 subtypes in ^V600E^*BRAF* mutated mCRC with a modern multivariate model including a validated clinical prognostic score as covariate. For each variable, we verified the impact on OS and secondarily on PFS. Moreover, level of concordance between primary tumours and metastatic sites was studied for each parameter.

Major findings were: CDX2 loss, high CK7 expression, less TILs, CMS2-3 or CMS4 (compared to CMS1) conferred worse OS. Progression-free survival analyses led to similar results. At multivariate analysis, CK7 and CMS subgrouping retained their significant correlation with OS.

CRC is usually associated to a CK7 negative and CK20/CDX2 positive profile.^12,13^ Notwithstanding, some evidence in literature suggests that in ^V600E^*BRAF* mutated mCRC this profile may be different, with a higher prevalence of CK20/CDX2 negative tumours.^14,15^ On the other hand, it has never been properly explored how the cytokeratins’ profile affects prognosis among *BRAF* mutated patients, moreover none of the survival analyses conducted so far included important covariates such as MSI status.^11^ In 2014 Landau et al. documented lower expression of CDX2 in ^V600E^*BRAF* mutated mCRC compared to ^V600E^*BRAF* wild type mCRC, irrespective of MSI status: these results suggest that loss of expression of CDX2 could depend on *BRAF* mutations more than on microsatellite status. Of note, in the same study a higher prevalence of CK7 expression was found in *BRAF* mutated MSS CRC. Unfortunately, no survival analyses were planned in that study.^25^ Recently, another work described the relationship between CDX2 and prognosis in CRC: in this study, loss of CDX2 expression was associated with significantly worse OS, but specific analyses for stage IV ^V600E^*BRAF* patients were not possible due to small sample size.^26^ According to our data, low CDX2 expression was associated to worse OS (HR of intermediate/low vs high expression = 1.72, 95%CI 1.03–2.86, *p* = 0.036).

We were also able to report on the prognostic role of CK7 expression, emerging as a strong determinant of outcome even in the multivariate model (HR of CK7 positive vs negative = 2.11, 95%CI 1.03–4–34, *p* = 0.041). From a mechanistic perspective, this finding is in line with what Harbaum et al. described earlier: a high prevalence of CK7 positive cells at the invasive front of tumour buds in samples of CRC. The same authors recorded a trend toward a higher risk of disease progression or mortality in patients with high CK7 expression.^27^ Again, no specific information was available regarding ^V600E^*BRAF* mutated patients in this study. The authors argued that CK7 could be associated with epithelial-to-mesenchymal transition. In our study we did not find significant correlations between CDX2/CK7/CK20 expression results and grading (Supplementary Table 5), nor with CMS classification (Supplementary Table 1).

In our study, CMS2-3 and 4 were associated with significantly worse OS and PFS (HR = 2.70, 95%CI 1.41–5.26, *p* = 0.003 and HR = 0.22, 95%CI 1.14–4.35, *p* = 0.02, respectively) when compared to CMS1 also in multivariate analyses. Given that we arbitrarily assigned MSI-H tumours to the CMS1 subgroup as previously described,^22,23^ our data clarify the effect of MSI-H phenotype on ^V600E^*BRAF* mutated mCRC. This finding provides an answer to an open issue still unsolved from studies conducted so far.^2,19^ It is unlikely that treatment with anti-PD1 could have influenced these results since the percentage of patients treated with those agents is below 10% in our series. Given the rapid development of anti-BRAF and immunotherapy with checkpoint inhibitor-based treatments in mCRC, our data would be quite useful to inform the design of future clinical trials by evaluating the adoption of new stratification factors. It should be noted that while IHC assessment for CMS subgrouping may lead to different results than standard transcriptome-based classification a concordance of nearly 90% was reported in specific studies^23^ and the intrinsic advantages of IHC in terms of costs and ease of reproducibility are obvious.

Another interesting observation is that presence of TILs was related to better OS and PFS outcome (respectively, HR = 0.58, 95%CI 0.39–0.86 for OS and HR = 0.58, 95%CI 0.39–0.85 for PFS). Recently, Shibutani et al. described a correlation between high immune infiltrate in primary tumour and response to chemotherapy in a cohort of 57 mCRC patients, with higher response rate to chemotherapy (79.3 vs. 48.1%, *p* = 0.025) and better PFS (10.1 months vs. 7.3 months, *p* = 0.013) in the high-TILs group. Of note, in the high-TILs group a significantly better OS was observed compared to low-TILs group (35.5 months vs. 22.4 months, *p* = 0.022).^28^ Taken together, data on TILs and CMS suggest a role for tumour-immune system interaction in affecting prognosis of ^V600E^*BRAF* mutated mCRC.

We also explored the reproducibility of BM1/BM2 categorisation with IHC based surrogate markers and correlated those results with outcome. Similarly, to what Barras et al. previously reported,^5^ no differences were found in PFS and OS between the two groups.

Major points of strength of our data rely on: (a) clinical homogeneity (i.e. all metastatic patients), (b) large numbers (^V600E^*BRAF* mutated mCRC constitutes around 8% and 155 patients with detailed clinical data and biologic material constitute one the biggest cohort ever studied), (c) adjustment with a modern, robust and validated clinical prognostic score, (d) real world data. The latter is a two-faced point, with its intrinsic pros and cons. From one side, being *BRAF* a determinant of extremely bad prognosis, it has been reported how patients with available biologic material or enrolled in trials do not resemble exactly in terms of incidence and specific characteristics. From this may obviously originate a dangerous selection bias when analysing patients and samples from trials.^1^ From the other side, we should admit that quality and detail of real-world data are certainly limited compared to prospective controlled trials. In fact, the most relevant limitation of our study is its retrospective nature. Patients have been selected according to stage, but not for type or number of previous lines of treatment received: in this way, a slight selection bias could not be excluded. Additional limitations reside in not having adjusted for multiple testing and not having enough biologic material for testing all the markers in the totality of the population included.

In conclusion, present data provide new, original and informative observations. These findings would deserve external confirmatory studies but give already a guide to interpret clinical heterogeneity within the subgroup of ^V600E^*BRAF* mCRC, being therefore useful to inform further translational research, for patients’ stratification in clinical trials and in routine clinical practice to better estimate patients’ prognosis.

## Supplementary information

Supplementary Tables and Figures

## Data Availability

The datasets used and/or analysed during the current study are available from the corresponding author on reasonable request.
