# Supplementary material for: CK7 and consensus molecular subtypes as major prognosticators in V600EBRAF mutated metastatic colorectal cancer
Source: Br J Cancer. 2019 Sep 2;121(7):593–9. doi: 10.1038/s41416-019-0560-0 (PMC6889398; doi:10.1038/s41416-019-0560-0)
Supplement: Supplementary file 1 — Supplementary Tables and Figures [file 41416_2019_560_MOESM1_ESM.docx]

**Supplementary Table 1. Correlation between paired single parameters in terms of percentage of positive cases out of total evaluable for both analyses.**

|  | **CK7+** | **CK20+** | **CMS1** | **CMS2-3** | **CMS4** | **TILs+** | **BM1** | **BM2** |
| --- | --- | --- | --- | --- | --- | --- | --- | --- |
| *CDX-2 intermediate* | 2.1% | 27.9% | 10.1% | 14.7% | 7.3% | 18.9% | 17.3% | 13.5% |
| *CDX-2*  *high* | 5.4% | 34.4% | 11.0% | 21.1% | 0.9% | 21.0% | 17.3% | 17.3% |
| *CK7+* |  | 10.7% | 4.3% | 5.4% | 3.2% | 6.4% | 6.5% | 5.4% |
| *CK20+* |  |  | 32.2% | 39.8% | 16.1% | 58.1% | 36.9% | 51.1% |
| *CMS1* |  |  |  |  |  | 37.8% | 9.7% | 25.2% |
| *CMS2-3* |  |  |  |  |  | 21.6% | 27.2% | 18.4% |
| *CMS4* |  |  |  |  |  | 6.3% | 11.6% | 7.7% |
| *TILs+* |  |  |  |  |  |  | 26.0% | 37.5% |

**Supplementary Table 2. Concordancy for primary/mets paired**

|  | | **TOT= 46 paired**  **N (%)** | ***Prim🡪 Mets***  ***N (%)*** | ***N (%)*** |
| --- | --- | --- | --- | --- |
| *CDX2* | Concordant | 41 (89.1%) |  |  |
|  | Discordant | 5 (10.9%) | Low🡪High | 1 (20%) |
|  |  |  | Int🡪Low | 3 (60%) |
|  |  |  | High🡪Low | 1 (20%) |
| *CK7* | Concordant | 46 (100%) |  |  |
|  | Discordant | 0 |  |  |
| *CK20* | Concordant | 46 (100%) |  |  |
|  | Discordant | 0 |  |  |
| *CMS* | Concordant | 35 (76.1%) |  |  |
|  | Discordant | 11 (23.9%) | CMS2-3🡪CMS4 | 5 (45.5%) |
|  |  |  | CMS4🡪CMS2-3 | 6 (54.5%) |
| *TILs* | Concordant | 45 (97.8%) |  |  |
|  | Discordant | 1 (2.2%) | Low🡪High | 1 (100%) |
| *BM* | Concordant | 20 (50%) |  |  |
|  | Discordant/NAs | 20 (50%) | BM1 border | 8 (40%) |
|  |  |  | BM2 border | 12 (60%) |
|  | *NE** | *6* |  |  |

** Not Evaluable*

**Supplementary Table 3. Univariate analysis for Progression-Free Survival**

| **Characteristics** | | **Median PFS (months)** | **Progression Free Survival** | | |
| --- | --- | --- | --- | --- | --- |
|  |  |  | **HR** | **95% CI** | **p** |
| *CDX2* | High | 9.6 | 1 | - | - |
|  | Intermediate | 5.8 | 1.41 | 0.86 – 2.30 | 0.169 |
|  | Low | 5.6 |  |  |  |
| *CK7* | Low | 8.5 | 1 | - | - |
|  | High | 4.3 | 1.13 | 0.56 – 2.29 | 0.740 |
| *CK20* | Pos | 8.0 | 1 | - |  |
|  | Neg | 3.5 | 1.72 | 0.73 – 4.05 | 0.213 |
| *CMS* | 1 | 11.0 | 1 | - | - |
|  | 2-3 | 7.0 | 2.22 | 1.14 – 4.35 | **0.020** |
|  | 4 | 8.0 |  |  |  |
| *TILs* | High | 8.9 | 1 | - | - |
|  | Low | 5.8 | 1.72 | 1.18– 2.56 | **0.005** |
| *BM* | 2 | 9.1 | 1 | - | - |
|  | 1 | 5.5 | 1.27 | 0.80 – 2.03 | 0.309 |
| *Simplified score* | Low | 9.0 | 1 | - | - |
|  | Intermediate | 8.0 |  |  |  |
|  | High | 3.4 | 2.13 | 1.21 – 3.75 | **0.009** |

**Supplementary Table 4. Multivariate analysis for Progression Free Survival**

| **Characteristics** | | **Progression Free Survival** | | |
| --- | --- | --- | --- | --- |
|  |  | **HR** | **95% CI** | **p** |
| *CMS* | CMS1 | 1 | - | - |
|  | CMS2-3 +CMS4 | 2.17 | 1.01–4.76 | **0.049** |
| *TILs* | High | 1 | - | - |
|  | Low | 1.16 | 0.67– 1.96 | 0.593 |
| *Simplified score* | Intermediate +Low | 1 | - | - |
|  | High | 2.52 | 1.25–5.11 | **0.010** |

**Supplementary Table 5. Distribution of Tumor Grading according to CDX2 and cytokeratins expression**

|  | | **G1-2** | **G3-4** |
| --- | --- | --- | --- |
| *CDX2* | Low | 13 (28%) | 34 (72%) |
|  | Intermediate | 14 (28%) | 36 (72%) |
|  | High | 13 (28%) | 34 (72%) |
| *CK7* | Low | 21 (26%) | 60 (74%) |
|  | High | 3 (25%) | 9 (75%) |
| *CK20* | Low | 3 (27%) | 8 (73%) |
|  | High | 21 (26%) | 61 (74%) |

**Supplementary Figure 1 – Kaplan-Meier curves for Progression-free survival (PFS).**


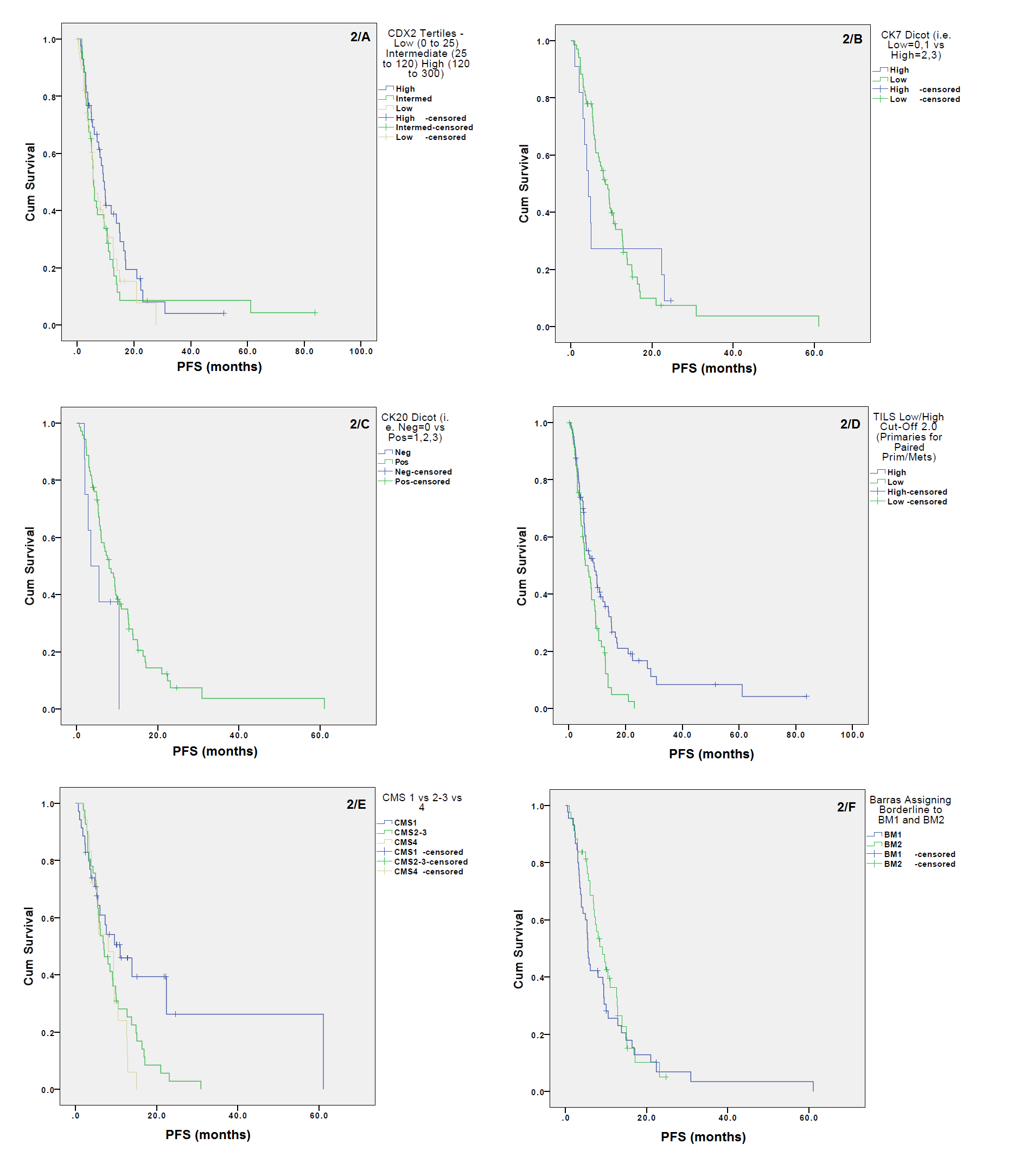


2/A - CDX2 tertiles expression (low vs intermediate and high)

2/B - CK7 expression (high vs low)

2/C - CK20 expression (negative vs positive)

2/D - TILs expression (low vs high)

2/E - CMS classification (CMS2/3 and CMS4 vs CMS1)

2/F - Barras classification (BM1 vs BM2)
